# Positive selection and comparative molecular evolution of reproductive proteins from New Zealand tree weta (Orthoptera, *Hemideina*)

**DOI:** 10.1371/journal.pone.0188147

**Published:** 2017-11-13

**Authors:** Victoria G. Twort, Alice B. Dennis, Duckchul Park, Kathryn F. Lomas, Richard D. Newcomb, Thomas R. Buckley

**Affiliations:** 1 School of Biological Sciences, The University of Auckland, Auckland, New Zealand; 2 Landcare Research, Auckland, New Zealand; 3 CSIRO, Melbourne, Australia; 4 The New Zealand Institute for Plant and Food Research Ltd, Auckland, New Zealand; USDA Agricultural Research Service, UNITED STATES

## Abstract

Animal reproductive proteins, especially those in the seminal fluid, have been shown to have higher levels of divergence than non-reproductive proteins and are often evolving adaptively. Seminal fluid proteins have been implicated in the formation of reproductive barriers between diverging lineages, and hence represent interesting candidates underlying speciation. RNA-seq was used to generate the first male reproductive transcriptome for the New Zealand tree weta species *Hemideina thoracica* and *H*. *crassidens*. We identified 865 putative reproductive associated proteins across both species, encompassing a diverse range of functional classes. Candidate gene sequencing of nine genes across three *Hemideina*, and two *Deinacrida* species suggests that *H*. *thoracica* has the highest levels of intraspecific genetic diversity. Non-monophyly was observed in the majority of sequenced genes indicating that either gene flow may be occurring between the species, or that reciprocal monophyly at these loci has yet to be attained. Evidence for positive selection was found for one lectin-related reproductive protein, with an overall omega of 7.65 and one site in particular being under strong positive selection. This candidate gene represents the first step in the identification of proteins underlying the evolutionary basis of weta reproduction and speciation.

## Introduction

Reproductive associated proteins have been shown to have increased evolutionary rates, and diverge rapidly between related taxa [1–6]. In particular, proteins present in the seminal fluid (SFPs) have been identified as often evolving under positive selection [4, 7–9]. In insects, the synthesis and secretion of SFPs occurs within male reproductive tract secretory tissues, such as accessory glands and testis [10, 11]. SFPs encompass a diverse range of functional classes and are involved in the modulation or induction of post mating responses in females [12–18]. In addition, SFPs have been identified as playing a key role in reproductive isolation between diverging lineages [2, 19–22].

Many *Drosophila* SFPs show increased evolutionary rates when compared to non-seminal proteins [1, 23, 24] and show evidence of positive selection [7–9, 25, 26]. However, not all SFPs exhibit rapid evolution; some show signs of evolutionary conservation [27–30], while others exhibit both conservation and rapid evolution in different regions of a single protein [31–33]. *Drosophila* SFPs have been shown to play a key role in reproductive isolation through species-specific gamete use, whereby SFPs need to have a specific structure and binding affinity for successful reproduction to occur [34]. Studies of orthopteran taxa have revealed similar patterns. In particular, studies on crickets have shown that SFPs have a higher level of divergence when compared with non-seminal proteins, with a significant proportion being under positive selection [7, 8, 25, 35]. Despite these studies in crickets, very little is known about SFP evolution in other orthopteran taxa.

Insects from the orthopteran family Anostostomatidae are collectively known in New Zealand by their Māori name, weta, and represent an important component of the native forest ecosystem [36]. All Tree (*Hemideina*) and Giant (*Deinacrida*) weta species are endemic to New Zealand, and include both relatively widespread and threatened species. The larger *Deinacrida* species have limited distributions and abundance, with 10 of the 11 species being under conservation management [37–40]. There are seven species of *Hemideina* distributed throughout New Zealand, most of which are abundant [37–41]. *Hemideina crassidens* (Blanchard) has the largest distribution of all weta species, with populations distributed in the south of the North Island, as well as in the north and west coast of the South Island in New Zealand [42]. *Hemideina crassidens* has two chromosomal races (15 and 19) that are morphologically identical and successfully produce offspring in laboratory crosses [43, 44]. *Hemideina thoracica* (White) is found in the upper three quarters of the North Island [39]. All populations are morphologically similar despite there being eight chromosomal races with diploid chromosome numbers ranging from 11 to 24 [45]. Individual populations have been shown to exhibit only a single karyotype, with interbreeding occurring in the narrow regions of contact [45–47]. The presence of multiple chromosomal races within these species indicates that chromosomal differences are insufficient to lead to reproductive isolation [45, 48]. *Hemideina trewicki* has the smallest distribution of the three species, occurring only in southern and central Hawke’s Bay. In the northern parts of the range, *H*. *trewicki* is sympatric with *H*. *thoracica* [42, 49] and has one known chromosomal race [48]. Although much progress has been made in revealing patterns of speciation and hybridisation in tree weta, the molecular basis of mate recognition, fertilisation and other reproductive processes are not known. The present study describes the male reproductive transcriptomes for *H*. *crassidens* and *H*. *thoracica*. We identify putative male reproductive associated proteins, and investigate patterns of divergence of nine genes between three *Hemideina* species. We test the hypothesis that male reproductive associated proteins have elevated rates of positive selection compared to general metabolic, or housekeeping, genes.

## Materials and methods

### Sample collection

*Hemideina thoracica*, *H*. *crassidens* and *Deinacrida mahoenui* (Gibbs) specimens were collected across their known distributions between 2010 and 2012 (S1 Table), by day and night searching. All samples were collected under a permit issued by the Department of Conservation (CA-31615-OTH). Insects were transported live to Landcare Research, Auckland, and then snap frozen and stored at -80°C.

### Transcriptome sequencing

Accessory gland and testis tissue from one adult male *H*. *thoracica* and *H*. *crassidens* (S1 Table) were dissected under a dissecting microscope in 100% ethanol. Total RNA was extracted from each tissue using TRIzol RNA extraction reagent (Life Technologies) according to the manufacturer’s protocol. A further RNA clean-up was performed using the RNeasy Mini Kit (Qiagen). RNA quality and quantity was determined using a Nanodrop (ThermoScientific) and an Agilent 2100 bioanalyzer (Agilent Technologies). High quality total RNA was used to synthesise cDNA using the SMARTer^TM^ PCR cDNA synthesis kit (Clontech) with a modified oligo-dT primer (Cap-TRSA-CV) [50]. Double stranded cDNA was purified using AMPure beads (Agencourt). Library quality was assessed using an Agilent 2100 bioanalyzer (Agilent Technologies) and quantified with the Quanti-i TM Picogreen® assay (Life Technologies). Cleaned cDNA was fed into the Rapid Library Preparation Protocol (Roche, GS Junior Titanium Series, June 2010) at the fragment end repair step, with each tissue sample being MID barcoded. The resulting libraries were pooled by tissue and sequenced in two runs on a 454 GS Junior (Roche) at Landcare Research (Auckland).

### Pre-processing, assembly and annotating RNA-seq data

Raw sequences were split by MID barcode using Geneious V5.4.6 [51]. Reads with ambiguous bases and low quality sequences were removed using SnoWhite 1.1.14 [52]. The primer and adaptor sequences were removed using CUTADAPT V1.1 [53]. Poly A/T tails longer than 15 bp from either end of the reads, and reads shorter than 50 bp were removed using PRINSEQ LITE V0.16 [54]. Cleaned reads were *de novo* assembled with Newbler GS *de novo* Assembler (V. 2.5.3), with default parameters, a minimum overlap of 25 bp and a minimum overlap identity of 95%. Redundancy in the alignment was removed using cd-hit-est V. 4.5.6 [55]. Poor *de novo* assembly of the *H*. *thoracica* dataset was observed, due to a lower sequencing quality of the testis library run. Therefore, in order to obtain a more representative dataset additional assembly steps were undertaken. The cleaned, trimmed reads from both *H*. *thoracica* libraries were reference assembled against the *H*. *crassidens* transcriptome using the Roche GS reference mapper (version 2.5.3, default parameters, except for a 25 bp overlap). The purpose of this reference assembly was to obtain contigs that were unassembled in the *de novo* assembly due to inadequate coverage. The reference and *de novo* assemblies were combined and subjected to a second round of redundancy removal with cd-hit-est. Preliminary tests on the combined assembly showed similar levels of blast homology, GO annotation and assembly statistics; therefore, this assembly replaced the *de novo* assembly for *H*. *thoracica* in downstream analyses. Singletons (unassembled reads) were excluded from downstream analysis. The assemblies were annotated using Blastx V2.6.0 [56] (e-value < 1e^-5^) against the GenBank non-redundant (nr) protein database (downloaded July 2017). Transcripts were searched for conserved protein domains with InterProScan [57] and GO terms were assigned using Blast2GO v2.8 [58]. Full-length transcripts were identified using Full-Lengther [59].

### Identification of reproductive associated, orthologous, and candidate genes

Orthologous genes were identified using a bidirectional best hit method, which has been shown to outperform more complex algorithms for orthology predication [60]. A pair-wise reciprocal blastn approach was carried out in Geneious (e-value threshold 1e^-3^) with orthologues being called if the best blast hit was identical in both directions.

Putative reproductive associated genes were identified in a two-step process. First, transcripts were identified based on mapping counts of *D*. *fallai* muscle RNA-seq reads downloaded from SRA (SRA accession: SRR5965744) using RSEM [61]. Second, transcripts unique to the reproductive transcriptomes in *H*. *thoracica* and *H*. *crassidens* were identified by mapping the *D*. *fallai* short reads to each assembly using Bowtie2 [62] and identifying the transcripts with no counts. Within these candidates, signal peptides, cellular location and the presence of trans-membrane domains were identified with SignalP (v4.1) [63], ProtComp v9.0 (http://linux1.softberry.com) and TMHMM v2.0 [64], respectively. Transcripts were retained as putative reproductive proteins if they had one of the following: (i) signal peptide, (ii) cellular localisation as extracellular and/or plasma membrane, (iii) transmembrane helix.

Candidate genes for downstream evolutionary analysis were chosen from among the contigs identified in the reproductive and orthologous gene screen. Candidates from the reproductive gene search were chosen based on their annotation, level of similarity (cut-off of 60%) and sequence length (minimum 400 bp). Orthologous candidates were based on a minimum transcript overlap of 200 bp between *H*. *thoracica* and *H*. *crassidens* transcripts, and the level of similarity at the amino acid and nucleotide level. Lastly, general metabolic control genes were chosen based on a minimum contig length of 300 bp and their involvement in general cellular processes, thereby ensuring tissue wide expression.

### Sequencing of candidate genes

For Sanger sequencing samples, total RNA extractions from testis tissue followed the methods described above for the RNA-seq samples. Contaminating DNA was removed from RNA extractions prior to cDNA synthesis using TURBO DNase (Invitrogen). The first strand cDNA synthesis used the SuperScript III First Strand Kit (Invitrogen) following the manufacturer’s protocol. cDNA libraries were subsequently amplified using 5 μL first strand cDNA, 0.8 μM random hexamer primer, 2X PCR buffer (Roche), 2.5 mM MgCl2 (Roche), 0.2 mM dNTP (Roche), 1 U FastStart Taq DNA polymerase (Roche) in a total volume of 53 μL. Amplifications were performed on a GeneAmp PCR system 9700 thermal cycler (Applied Biosystems) using the following parameters: 5 min at 95°C, 3 min at 50°C, 40 sec 72°C; 40 cycles of 40 sec at 94°C, 40 sec at 65°C and 40 sec at 72°C; and 10 min at 72°C.

Primers for nine candidate genes (*COI*, *Protease*, *Sflag*, *EFdelta*, *Unk2*, *Tkinase*, *Acp3*, *Acp4*, *Acp5*) were designed using Primer 3 [65] implemented within Geneious. Primer pairs were designed to amplify products of 200–1500 bp in length, have TMs of 60°C (±3°C) and to have a GC continent of 40–60% (S2 Table). Target genes were PCR amplified with reactions consisting of approximately 5 ng DNA, 1X PCR buffer (Roche), 2 mM MgCl2 (Roche), 0.2 mM dNTP (Roche), 0.1–0.2 μM forward and reverse primers (Sigma-Aldrich), 1 U FastStart Taq DNA polymerase (Roche), in a total volume of 25 μl. Amplification were performed on a GeneAmp PCR system 9700 thermal cycler (Applied Biosystems) using the following parameters: 5 mins at 95°C; 40 cycles of 15 sec at 95°C, 30 sec at primer specific annealing temperature and 1 min 30 sec at 72°C; and 5 min at 72°C.

PCR products were sequenced using BigDye Terminator Cycle Sequencing Ready Reaction Mix v3.1 (Applied Biosystems). Cycle sequencing products were cleaned using the BigDye Xterminator Purification Kit (Applied Biosystems) and sequenced in both directions on the ABI Prism 3100 Genetic Analyzer (Applied Biosystems). Sequences were subsequently cleaned, trimmed and aligned using Geneious. In addition, the *D*. *mahoenui* and *D*. *fallai* transcriptomes (unpublished) were searched for *COI* and all candidate gene orthologues, respectively using a bidirectional tblastx approach, and included in downstream analysis (sequences given in S1 File).

Haplotype reconstruction for sequences that exhibited heterozygosity was performed using PHASE V. 2.1 [66, 67] prior to calculating descriptive statistics in DnaSP V. 5 [68]. Tajima’s D [69] and the McDonald-Kreitman test [70] were calculated in DNAsp. Haplotype networks were constructed using TCS V. 1.2.1 (Clement, Posada, Crandall 200), with gaps being considered as the 5^th^ state and a 95% connection limit. The substitution model for the *COI* phylogeny was selected using the corrected Akaike information criterion [71] generated by jModel Test v.0.0.1 [72, 73]. A maximum likelihood phylogeny was constructed in Garli v2.0 [74] using 100 search and 1000 bootstrap replicates.

### Inferring positive selection

Genes were identified for selection tests based on the number of non-synonymous changes. The three candidates with the most non-synonymous changes (*Acp3*, *Protease*, *Unk2)* were chosen for downstream analysis. For these three, a neighbour-joining phylogeny was generated for selection tests in Geneious. To screen for positive selection ω was estimated by maximum likelihood, using codon-based substitution models implemented in the CODEML package of PAML V. 4.5 [75]. The models implemented (M0, M1a, M2a, M3, M7, M8, M8a) are extensively described elsewhere [76–78]. Complex models (M2a, M3, M8) allow more than one category of ω, thereby allowing individual codons to be identified as under positive selection when the average ω across the whole gene indicates purifying selection. Likelihood ration tests (LRTs) between nested models allows inference of positive selection acting on a sequence [75]. Codons under positive selection were identified using the Bayes empirical Bayes (BEB) method under the M8 model.

## Results and discussion

### Transcriptome assembly and characterisation

**Fig 1:**
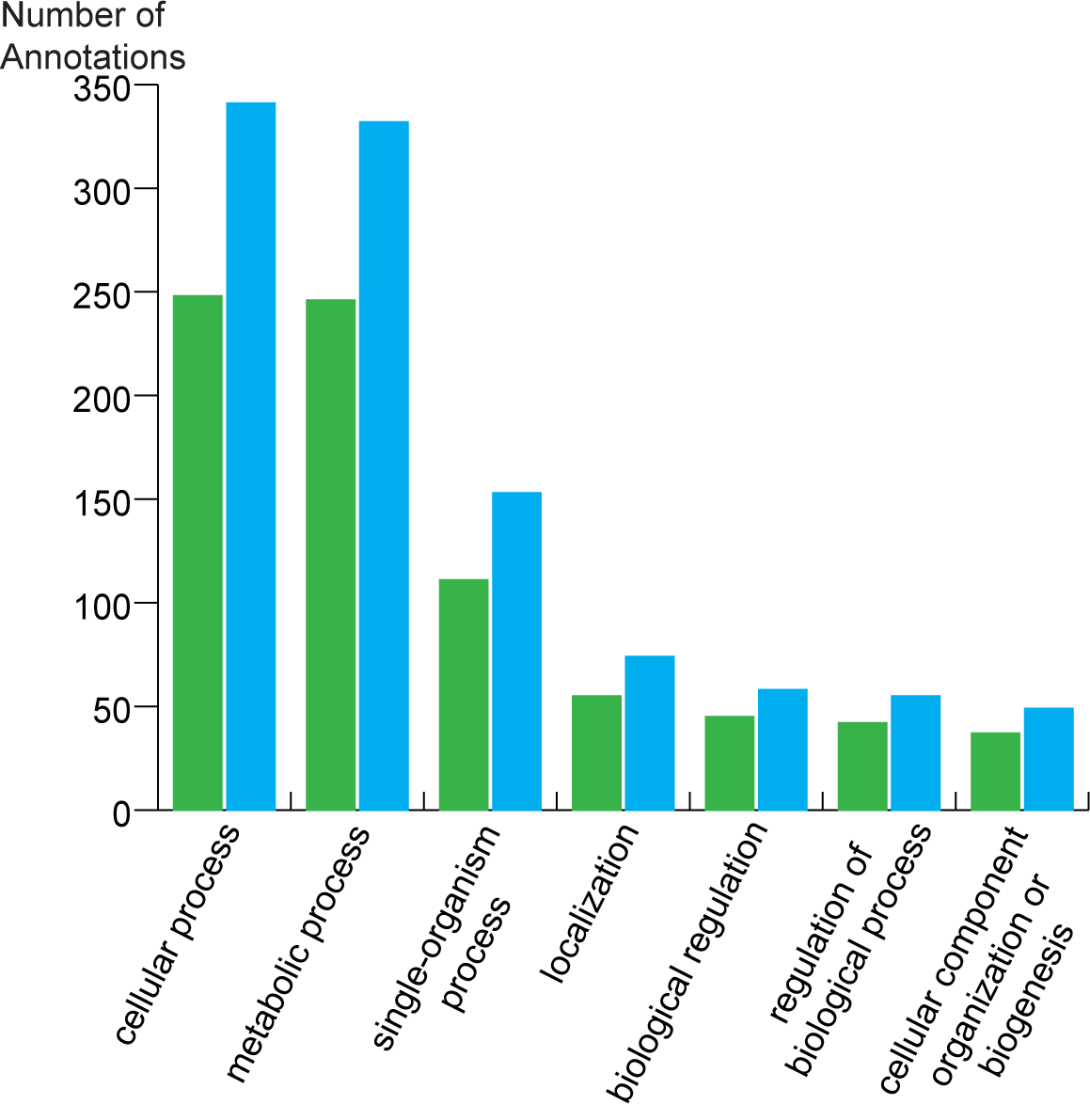
Distribution of biological function annotation of two *Hemideina* transcriptomes. Green bars: *H*. *crassidens* and blue bars: *H*. *thoracica*.

454 sequencing of *H*. *thoracica* and *H*. *crassidens* cDNA libraries resulted in a total of 254,628 reads, of which 73,012 and 59,465 reads were from *H*. *thoracica*, and 37,384 and 84,767 from *H*. *crassidens* testis and accessory gland tissue libraries, respectively. Raw sequences have been submitted to the GenBank Short Read Archive (BioProject: PRJNA353021). After trimming to remove bases with low quality scores, adapters and MID barcodes, 97% of the data remained. *De novo* assemblies were generated for each species as described above. The *H*. *crassidens* assembly generated 1,759 unigenes with an N50 of 608 bp and a maximum transcript size of 2,861 bp. In comparison, the *H*. *thoracica* assembly generated 2,691 unigenes with an N50 of 576 bp and a maximum transcript size of 1,860 bp. Both assemblies have been submitted to TSA under GFBX00000000 and GFBW00000000. A total of 890 and 1,537 transcripts were identified as being full length, respectively. Approximately 45% of the unigenes present in each assembly were functionally annotated using a tblastx search against the NCBI non-redundant database, with the species distribution of top matches overlapping among the two assemblies (S1 Fig). Functional annotation (GO) was similar across the two species, with the highest number of annotated transcripts related to cellular processes (GO:0009987) and metabolic process (GO:000812) (Fig 1). All unigenes were screened against the InterPro database, from which 2,690 and 1,753 *H*. *thoracica* and *H*. *crassidens* transcripts, respectively, were identified as containing conserved protein domains. The top 20 most frequent entries are shown in Table 1. These top entries show a diverse range of predicted functions, including proteins associated with general house-keeping roles and other that have been linked to reproductive functions. Domains identified include ubiquitin (IPR000626, IPR029071) and translation protein (IPR008991) domains. Many of the genes represented in these groups are probably highly conserved genes involved in the processes of transcription and protein degradation. Other domains identified, such as proteases (IPR001254, IPR018114) and protease inhibitors (IPR00215, IPR023796), have been associated with a number of reproductive functions, such as the modifying postmating changes in females [79], and are frequently identified in the study of insect SFPs [9, 80, 81].

**Table 1:** The 20 most encountered InterPro accessions present in two *Hemideina* transcriptomes.

*H*. *thoracica*			*H*. *crassidens*		
InterPro Entry	InterPro Description	Number of Contigs	InterPro Entry	InterPro Description	Number of Contigs
IPR012336	Thioredoxin-like fold	23	IPR016187	C-type lectin fold	17
IPR011991	Winged helix-turn-helix DNA-binding	18	IPR016186	C-type lectin-like/link domain	16
IPR029071	Ubiquitin-related domain	17	IPR001304	C-type lectin-like	14
IPR027417	P-loop containing nucleoside triphosphate	17	IPR012336	Thioredoxin-like fold	13
IPR000504	RNA recognition motif domain	15	IPR001254	Serine proteases, trypsin domain	13
IPR000626	Ubiquitin domain	15	IPR009003	Peptidase S1, PA clan	13
IPR016187	C-type lectin fold	12	IPR011991	Winged helix-turn-helix DNA-binding	10
IPR010987	Glutathione S-transferase, C-terminal-like	12	IPR011992	EF-hand-like domain	9
IPR014756	Immunoglobulin E-set	11	IPR023796	Serpin domain	8
IPR015943	WD40/YVTN repeat-like-containing domain	11	IPR027417	P-loop containing nucleoside triphosphate	8
IPR016186	C-type lectin-like/link domain	11	IPR029277	Single domain Von Willebrand factor type C domain	8
IPR011992	EF-hand domain pair	10	IPR013783	Immunoglobulin-like fold	7
IPR004045	Glutathione S-transferase, N-terminal	10	IPR008037	Pacifastin domain	7
IPR000477	Reverse transcriptase domain	10	IPR000477	Reverse transcriptase domain	7
IPR032675	Leucine-rich repeat domain, L domain-like	9	IPR002048	EF-hand domain	7
IPR008991	Translation protein SH3-like domain	9	IPR029071	Ubiquitin-related domain	7
IPR005203	Hemocyanin, C-terminal	9	IPR016040	NAD(P)-binding domain	7
IPR001304	C-type lectin-like	9	IPR000626	Ubiquitin domain	6
IPR016040	NAD(P)-binding domain	8	IPR007110	Immunoglobulin-like domain	6
IPR013766	Thioredoxin domain	7	IPR013766	Thioredoxin domain	6

### Identification of orthologous transcripts and reproductive proteins

The bidirectional best hit method identified 113 pairs of sequences that were putatively orthologous between the two species (S3 Table). For simplicity, these genes are hereafter referred to as orthologous. Some orthologues will have been missed using this approach, as in *D*. *melanogaster* the evolutionary rate of some SFPs has been shown to be so rapid that they lack any detectable similarity with their homologues from other *Drosophila* species [1, 24, 82, 83].

Putative reproductive associated transcripts were identified by mapping *D*. *fallai* muscle RNA-seq reads to each transcriptome. Transcripts unique to the reproductive transcriptomes (those lacking mapped reads) were further analysed to identify putative reproductive associated proteins. Of the transcripts unique to the reproductive transcriptome, 258 and 337 meet the criteria of having a signal peptide, transmembrane helix or localisation at the plasma membrane or extracellular for *H*. *thoracica* and *H*. *crassidens*, respectively (S4 Table). Roughly 19% of these had Blast hits, indicating that those lacking homology might be novel proteins or proteins highly diverged in weta. Among the genes with Blast hits, the most common molecular function GO terms were serine-type endopeptidase activity (GO:0004252), serine-type endopeptidase inhibitor activity (GO:0004867) and ATP binding (GO:0005524) (S2 Fig). Overall, the GO categories identified in the reproductive gene search are similar to categories commonly seen when studying insect SFPs [9, 16, 81, 84]. Various peptidase and peptidase regulators are among the reproductive proteins identified, and are believed to be essential for the regulation of reproduction through proteolytic cascades [28]. These types of proteins constitute a large proportion of the *D*. *melanogaster* [80], *Anopheles gambiae* [85], *Aedes aegypti* [86], *Lutzomyia longipalphis* [87] and *Clitarchus hookeri* [84] identified SFP and accessory gland proteins. The reproductive proteins identified in this screen are similar when compared with other insects, however a large proportion lack Blast hits. These unknown transcripts indicate the presence of novel or highly divergent proteins, and provide a large resource for the study of sexual reproduction and speciation in the New Zealand weta [80, 85–90].

### Candidate gene identification and sequencing

To study the patterns of molecular evolution of weta reproductive proteins, alignments of the candidates generated from the transcriptome sequencing were used to design PCR primers from nine genes (Table 2). Six putative reproductive proteins (*Acp3*, *Acp4*, *Acp5*, *Sflag*, *Tkinase*, and *Protease*) were identified as interesting candidates for downstream evolutionary analysis based on their blast homologies. The contig *Unk2*, despite lacking significant blast homology, was included for further analysis based on the interesting amino-acid pairwise identity observed during the orthologue gene screen. In addition, one nuclear (*EFdelta*) and one mitochondrial (*COI*) gene were included as general metabolic controls due to their tissue-wide expression. All nine genes were successfully amplified and sequenced from cDNA from, 19 *H*. *thoracica*, 11 *H*. *crassidens*, *5 H*. *trewicki* and 1 *D*. *mahoenui* (outgroup) individuals. In addition, transcripts were identified within our unpublished RNA-seq data for *D*. *fallai* for all nine genes. All sequences have been submitted to NCBI GenBank (accession numbers: KY999988—KY999999, MF000001—MF000301).

**Table 2:** Candidate genes for downstream evolutionary analysis.

	Gene	Annotation	*H*. *thoracica*	*H*. *crassidens*	AA % identity	Nuc % identity
General metabolic controls	*COI*	Cytochrome oxidase subunit I	contig01355	contig00784	80.2	81.3
	*Efdelta*	Elongation factor 1 delta	refmapcontig00958	contig00992	99.2	97.5
Reproductive proteins	*Protease*	Serine protease snake-like	contig02289	contig01653	90	94.1
	*Tkinase*	Testis specific ser/thr kinase	contig01847	—	—	—
	*Sflag*	Sperm flagellar protein	contig01892	—	—	—
					
	*Acp5*	Accessory gland protein	refmapcontig01086	contig00834	93.5	98.1
	*Acp4*	Accessory gland protein	refmapcontig00651	contig01386	90.4	97.3
	*Acp3*	Accessory gland protein	refmapcontig00312	contig01836	88.5	90.8
	*Unk2*	—N/A—	refmapcontig00670	contig1351	93.8	93.7

AA, amino acid

Nuc, nucleotide

### Polymorphism, divergence and molecular evolution

**Fig 2:**
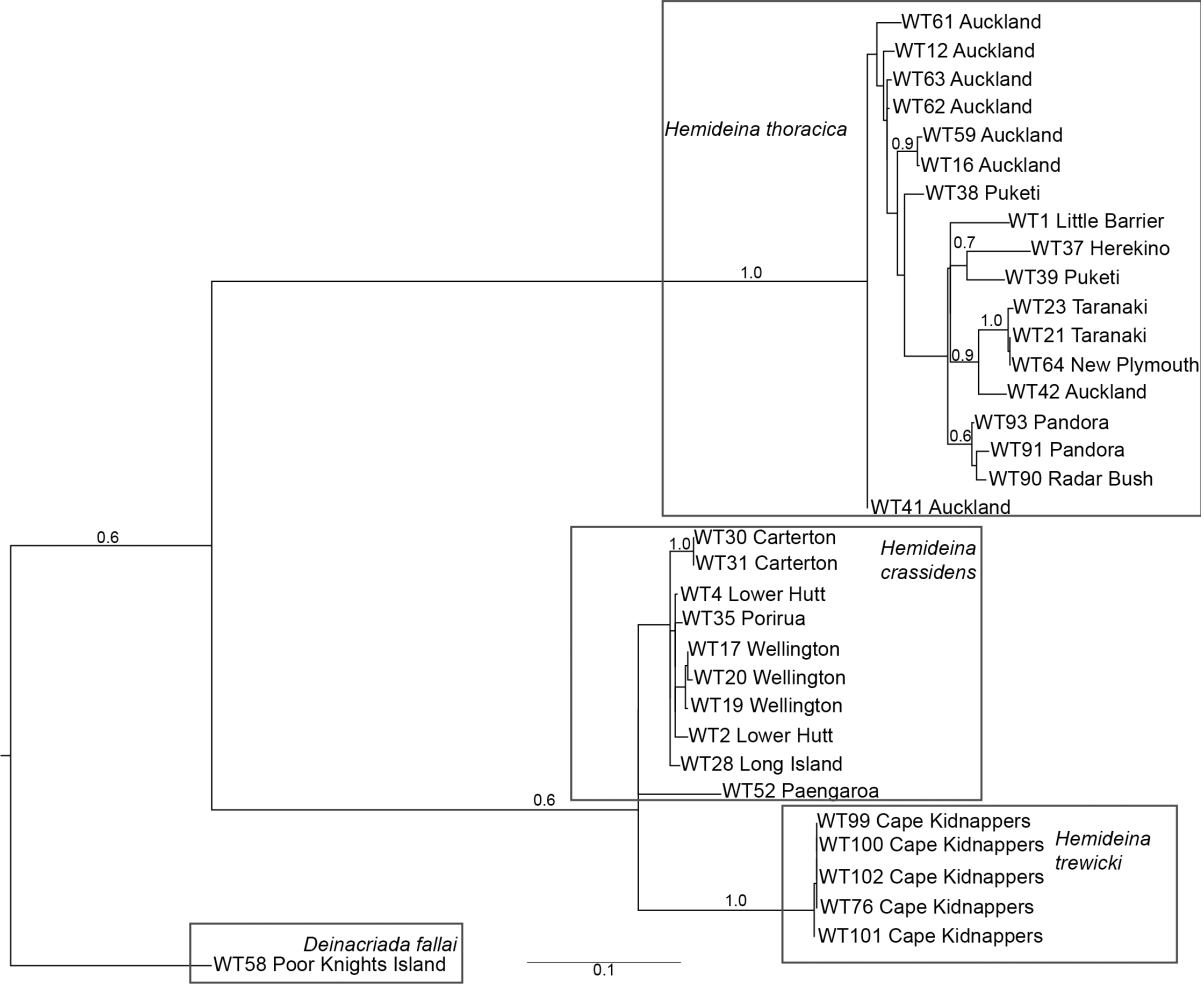
Maximum likelihood phylogeny constructed using mitochondrial cytochrome oxidase subunit I (*COI)* DNA sequences from individuals representing three *Hemideina* and one *Deinacrida* species. Bootstrap support values greater than 0.5 are indicated. Scale bar represents the number of substitutions per site.

Sequence data was obtained for *COI* from the majority of *Hemideina* individuals, resulting in a 672 bp alignment. The maximum likelihood phylogeny (Fig 2) supports each *Hemideina* species as monophyletic, with *H*. *trewicki* being sister to *H*. *crassidens*. The pairing of *H*. *crassidens* with *H*. *trewicki* is consistent with previous genetic and allozyme studies [41, 91].

**Fig 3:**
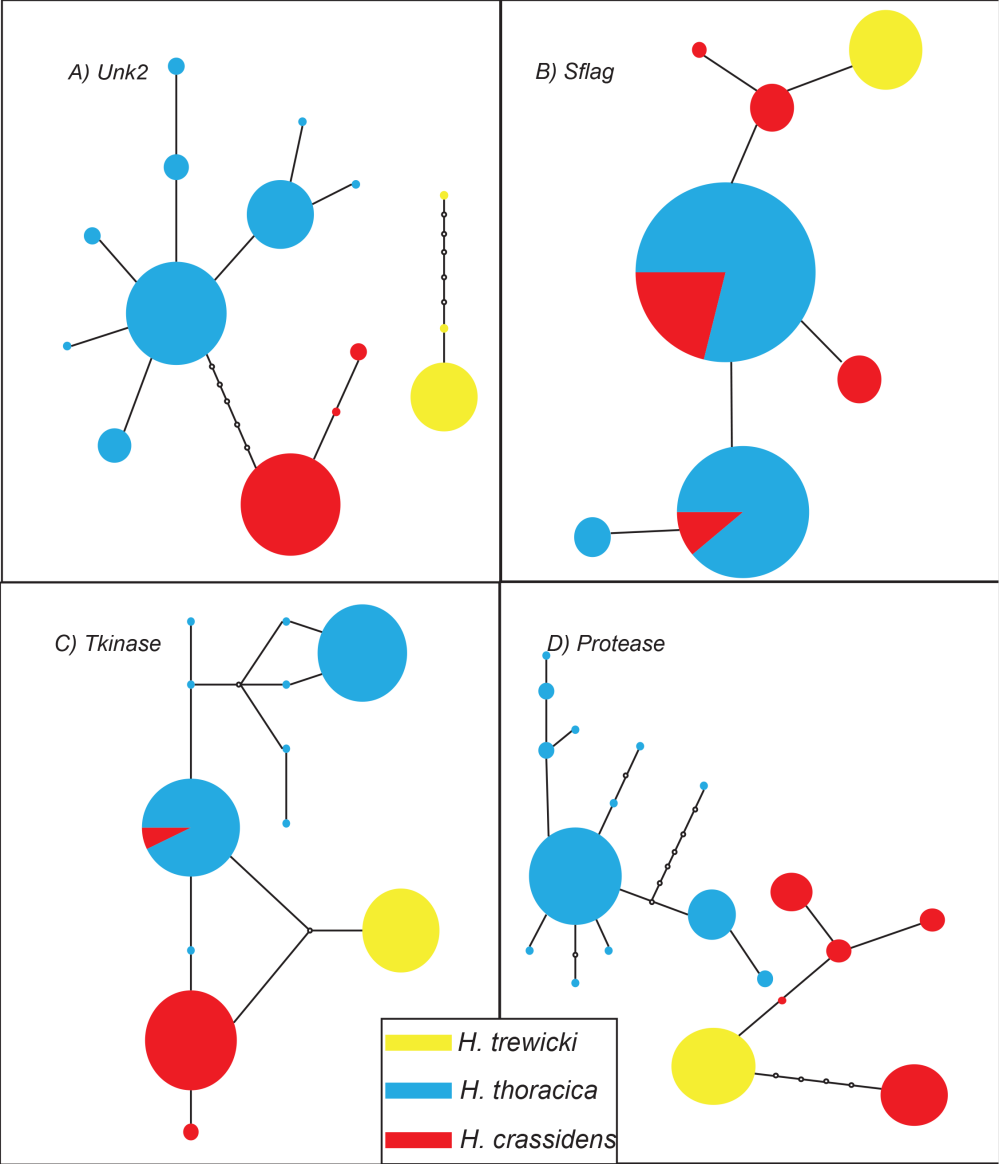
**Haplotype network of A) *Unk2*, B) *Sflag*, C) *Tkinase*, and D) *Protease* gene regions.** Circles represent different haplotypes, with the circles area being proportional to the frequency of each haplotype. Lines between haplotypes represent mutational steps between sequences. The empty circles represent inferred unsampled haplotypes. Colours correspond to species: Red, *H*. *crassidens*; Blue, *H*. *thoracica*; Yellow, *H*. *trewicki*.

**Fig 4:**
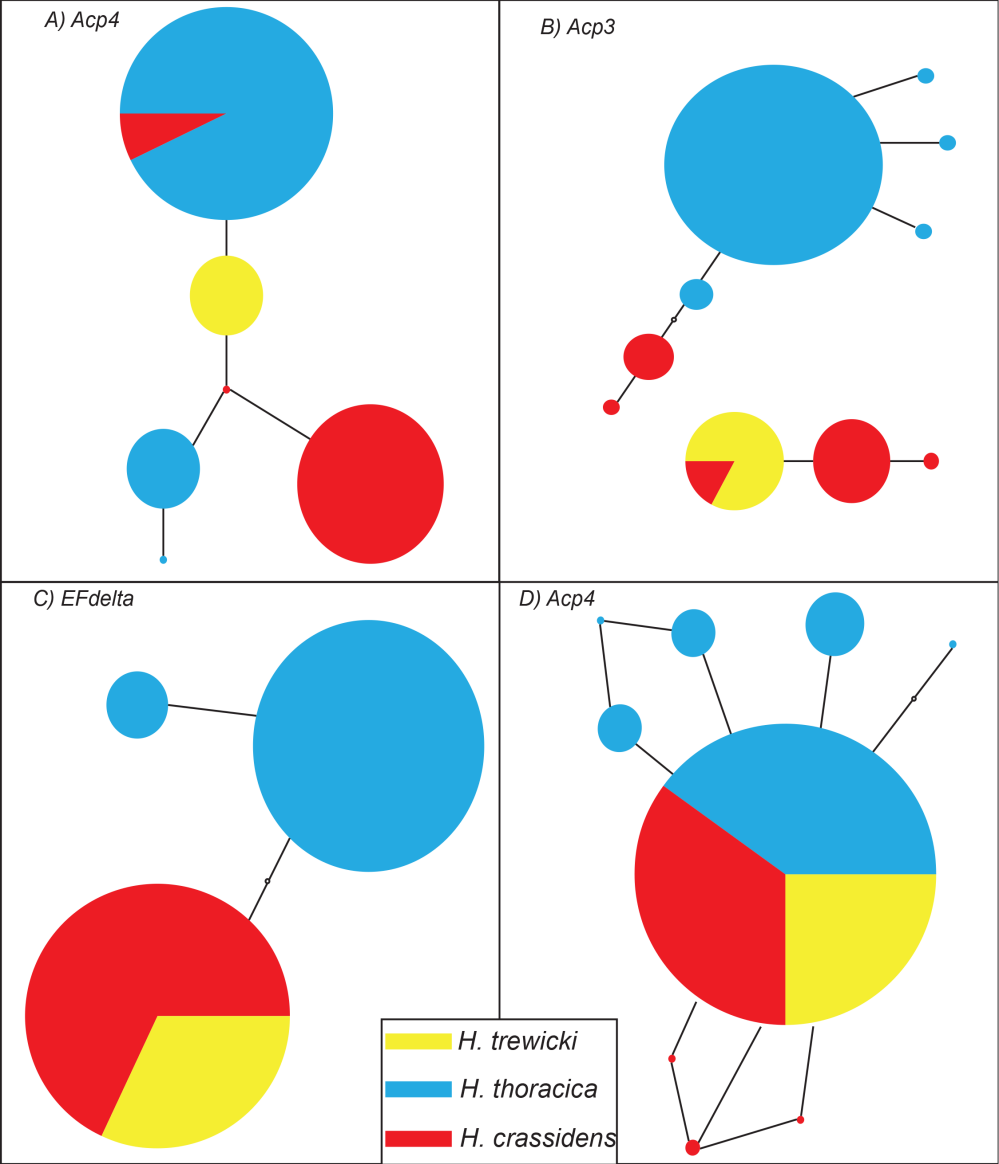
**Haplotype network of A) *Acp4*, B) *Acp3*, C) *EFdelta*, and D) *Acp5* gene regions**. Circles represent different haplotypes, with the circles area being proportional to the frequency of each haplotype. Lines between haplotypes represent mutational steps between sequences. The empty circles represent inferred unsampled haplotypes. Colours correspond to species: Red, *H*. *crassidens*; Blue, *H*. *thoracica*; Yellow, *H*. *trewicki*.

Haplotype networks were constructed for every gene, except *COI*, rather than phylogenetic trees. Very little sequence divergence was present within these genes, thereby reducing the statistical power of phylogenetic reconstruction [92, 93]. Two genes (*Unk2*, *Protease*, Fig 3A and 3D) showed monophyletic groupings of alleles, whereas the remaining six genes showed the presence of shared alleles between at least two of the species sequenced (Figs 3 and 4). Generally speaking, both the reproductive and general metabolic control genes showed similar patterns. Previous work has shown that at both a genetic [39, 94] and karyotypic [48] level *H*. *crassidens and H*. *trewicki* are more genetically similar to each other than either is to *H*. *thoracica*, and hence are more likely to produce fertile hybrids. Of the 8 genes sequenced, only *Protease* and *Unk2* show a complete lack of allele sharing among the species. The genes *Tkinase*, *Sflag*, *Acp4*, and *Acp5*, show sharing of alleles between *H*. *thoracica* and *H*. *crassidens*. The geographically restricted *H*. *trewicki* shares alleles with *H*. *crassidens* (*EFdelta*, *Acp3*, *Acp5*) and *H*. *thoracica* (*Acp5*). The two *Deinacrida* species are well differentiated from the three sampled *Hemideina* species at all loci, in agreement with previous studies [39, 91]. Overall these results demonstrate genetic differentiation among the three tree weta species, in agreement with McKean *et al*. [48].

A summary of intraspecific sequence variation is shown in Table 3. Intraspecific variation within *H*. *thoracica* was greater than that observed in *H*. *crassidens* and *H*. *trewicki* for the majority of genes examined. This is consistent with allozyme and mitochondrial DNA studies that show *H*. *thoracica* has the highest levels of intraspecific diversity of all *Hemideina* species [39, 41, 91]. This signature is consistent with inferences that the range of *H*. *thoracica* has recently expanded southwards [46, 95],while in comparison, little to no diversity was observed within the *H*. *trewicki* samples. This is not an unexpected result as all samples originated from a single population.

**Table 3:** Summary statistics of intra-specific sequence variation within three *Hemideina* species.

	Gene	Species	N	n	s	H	Hd	Eta	S	π	k	π_s_	π_a_	πa/π_s_	S	NS
Reproductive proteins	*Protease*	*H*. *thoracica*	18	36	423	13	0.798	19	19	0.005	2.175	0.008	0.004	0.5	8	11
		*H*. *crassidens*	10	20	423	5	0.768	8	8	0.008	3.711	0.118	0.007	0.0593	3	5
		*H*. *trewicki*	5	10	423	1	0.000	0	0	0.000	0.000	0.000	0.000	—	0	0
	*Tkinase*	*H*. *thoracica*	19	38	438	9	0.680	8	7	0.005	2.241	0.015	0.002	0.133	5	3
		*H*. *crassidens*	11	22	438	3	0.255	3	3	0.001	0.355	0.002	0.000	0.000	2	1
		*H*. *trewicki*	5	10	438	1	0.000	0	0	0.000	0.000	0.000	0.000	—	0	0
	*Sflag*	*H*. *thoracica*	19	38	333	3	0.619	2	2	0.002	0.747	0.010	0.000	0.000	2	0
		*H*. *crassidens*	11	22	333	5	0.797	5	5	0.005	1.662	0.014	0.002	0.142	3	2
		*H*. *trewicki*	5	10	333	1	0.000	0	0	0.000	0.000	0.000	0.000	—	0	0
	*Acp3*	*H*. *thoracica*	18	36	258	5	0.470	4	4	0.002	0.527	0.000	0.003	—	0	4
		*H*. *crassidens*	11	22	258	5	0.727	12	11	0.020	4.762	0.031	0.017	0.548	4	8
		*H*. *trewicki*	5	10	234	1	0.000	0	0	0.000	0.000	0.000	0.000	—	0	0
	*Acp4*	*H*. *thoracica*	19	38	237	3	0.437	3	3	0.005	1.24	0.017	0.002	0.118	2	1
		*H*. *crassidens*	11	22	237	3	0.177	3	3	0.001	0.355	0.005	0.000	0.000	2	1
		*H*. *trewicki*	5	10	237	1	0.000	0	0	0.000	0.000	0.000	0.000	—	0	0
	*Acp5*	*H*. *thoracica*	18	36	459	6	0.763	5	5	0.002	1.111	0.011	0.000	0.000	5	0
		*H*. *crassidens*	10	20	459	4	0.363	3	1	0.001	0.363	0.004	0.000	0.000	3	0
		*H*. *trewicki*	5	10	459	1	0.000	0	0	0.000	0.000	0.000	0.000	—	0	0
	*Unk2*	*H*. *thoracica*	17	34	372	9	0.813	8	7	0.004	1.303	0.005	0.003	0.600	3	5
		*H*. *crassidens*	11	22	372	3	0.279	2	2	0.001	0.458	0.000	0.002	—	0	2
		*H*. *trewicki*	5	10	372	3	0.378	7	7	0.004	1.556	0.008	0.003	0.375	3	4
General metabolic controls	*EFdelta*	*H*. *thoracica*	19	38	363	2	0.341	1	1	0.001	0.341	0.000	0.001	—	0	1
		*H*. *crassidens*	11	22	363	5	0.644	8	8	0.004	1.608	0.000	0.000	—	0	0
		*H*. *trewicki*	5	10	363	1	0.000	0	0	0.000	0.000	0.000	0.000	—	0	0
	*COI*	*H*. *thoracica*	18	18	672	17	0.993	134	119	0.050	34.22	—	—	—	—	—
		*H*. *crassidens*	10	10	672	9	0.978	60	59	0.024	16.33	—	—	—	—	—
		*H*. *trewicki*	5	5	672	2	0.400	1	1	0.001	0.400	—	—	—	—	—

N, number of individuals; n, number of alleles; s, number of sites; H, number of haplotypes; Hd, haplotype diversity; Eta, number of mutations; S, number of segregating sites; π, nucleotide diversity; k, average number of nucleotide differences; πs, nucleotide diversity at synonymous sites; πa, nucleotide diversity at nonsynonymous sties; S, total number of synonymous substitutions; NS, total number of nonsynonymous substitutions

The reproductive-associated candidate genes tended to display higher levels of within species diversity than the general metabolic controls. However, in some reproductive genes, especially *Acp5* and *Tkinase*, the observed level of genetic diversity was at the same or similar levels as the general metabolic controls. The relatively lower levels of diversity in these two reproductive genes suggest they are functionally constrained. However, this requires further investigation, as only two metabolic controls were included in this study, and only partial transcripts were sequenced. Possible explanations for the lower diversity, include the sequenced region may be in a functionally constrained region of the protein, with relaxed selection occurring upstream or downstream of the sequenced region, or these genes may be located in regions of low recombination.

Within the *Acp3* alignment an allelic variant containing a 24 bp indel was identified. All *H*. *thoracica* and two *H*. *crassidens* individuals have the insertion, while the remainder of *H*. *crassidens* and all *H*. *trewicki* samples lack the insertion. The 24 bp indel, appears to be a true allelic variant rather than the effect of preferential amplification of a paralogous gene as some individuals had only one copy of either the deletion or complete variant. If the deletion variant was in fact paralogous amplification then both copies would have been expected in all individuals of *H*. *crassidens*. InterProScan analysis revealed the presence of C-lectin type domains within the coding sequence. Lectins and lectin-related proteins have been shown to be involved in carbohydrate binding and the mediation of sperm-egg interactions [88–90], suggesting that this is an interesting reproductive candidate gene family.

At the species level, Tajima’s D was significant for the serine protease gene (*Protease*) within *H*. *crassidens*, (D = 2.17, Table 4) which may indicate balancing selection or demographic influences. In addition, for *Acp4*, *Acp3*, *Acp5*, *Unk2*, *and Tkinase* Tajima’s D statistics were negative for at least one species, thereby indicating an excess of rare or recent mutations that may be due to purifying selection or a recent demographic expansion, the latter of which has been observed for *H*. *crassidens* and *H*. *thoracica* [95]. Under the McDonald-Kreitman test no departures from neutrality were detected for any of the genes (S5 Table).

**Table 4:** Tajima’s D test results for three *Hemideina* gene *datasets*.

Gene	Species	D-statistic
*Protease*	*H*. *thoracica*	2.175*
	*H*. *crassidens*	-1.759
	*H*. *trewicki*	—
*Tkinase*	*H*. *thoracica*	0.511
	*H*. *crassidens*	-1.471
	*H*. *trewicki*	—
*EFdelta*	*H*. *thoracica*	0.629
	*H*. *crassidens*	—
	*H*. *trewicki*	—
*Acp3*	*H*. *thoracica*	-1.111
	*H*. *crassidens*	1.564
	*H*. *trewicki*	—
*Acp4*	*H*. *thoracica*	0.637
	*H*. *crassidens*	-1.471
	*H*. *trewicki*	—
*Acp5*	*H*. *thoracica*	-0.240
	*H*. *crassidens*	-1.529
	*H*. *trewicki*	—
*Unk2*	*H*. *thoracica*	-0.913
	*H*. *crassidens*	-0.440
	*H*. *trewicki*	-1.573

*, p-value < 0.05.

To study the patterns of molecular evolution of weta reproductive proteins, the ratio of nonsynonymous to synonymous substitution rates of protein-coding sequences (ω) was calculated for two reproductive genes (*Acp3*, *Protease*) and the unknown gene, *Unk2*. The candidate *Unk2* was included as part of the selection tests, as initial screening identified an ORF, which showed higher numbers of nonsynonymous than synonymous substitutions (Table 3). In the case of *Acp3* the mean ω ratio was >1, indicating an excess of nonsynonymous changes across the protein coding region as a whole (Table 5). In contrast, *Unk2* and *Protease* had an ω <1 (Table 5). Omega ratios averaged over an entire protein coding region are typically <1, due to positive selection commonly acting on specific domains or residues [96], with evidence suggesting genes with mean ω ratios above 0.5 are experiencing episodes of adaptive evolution [1, 7, 97, 98]. Individual amino acid residues likely to have been influenced by selection were identified using site-based ω calculations, with likelihood ratio tests and chi-squared distributions used to assess the goodness of fit for a given model [77, 99, 100]. For comparison, we used six codon-substitution models to assess the mode of selection acting on each amino acid residue in our candidate genes. We found that for *Acp3* and *Protease* there is significant among-site variation in ω, with the M3 model permitting three ω values providing a significantly better fit to the data than the M0 model (p-value<0.05; df = 4; M0:M3; Table 5, S6 Table). The M1a:M2a comparison was insignificant for all three genes. A more conservative approach for testing for positive selection is the M8:M8a comparison, under this model *Acp3* was identified was being under positive selection with a mean ω of 7.9 (Tables 5 and S6).

**Table 5:** Likelihood ratio tests of positive section using PAML site-specific models.

Gene (#sequences)	dN/dS	2Δl M0:M3	2Δl M1a:M2a	2Δl M8:M8a	Positive Selection (%)
*Acp3* (72)	1.11	10.62*	7.65	7.65*	6.8 (1.4)
*Prot* (70)	0.51	10.57*	0.00	1.20	11–47 (0–47)
*Unk2* (68)	0.72	1.22	0.17	0.17	4.4–16 (0)

dN/dS is the average omega (ω) across all sites and branches calculated under M0. 2Δl is given for each model comparison (M0:M3; M1a:M2a; M8:M8a), which is twice the difference between the log likelihood of the two nested site-specific models implemented in PAML. Models are judged to have a significantly better fit (* = P-value<0.05) based on the chi2 distribution with degrees of freedom proportional to the difference in the number of parameters between models; M0:M3 = 4; M1a:M2a = 4; M8:M8a = 50:50 mixture of point mass 0 and 1).

Parameters indicating positive selection are in bold. Percent positive selection indicates the proportion of sites across the gene predicted to have experienced positive selection, while the percentage in bracketed represents the proportion of sites identified with a >95% probability.

**Fig 5:**
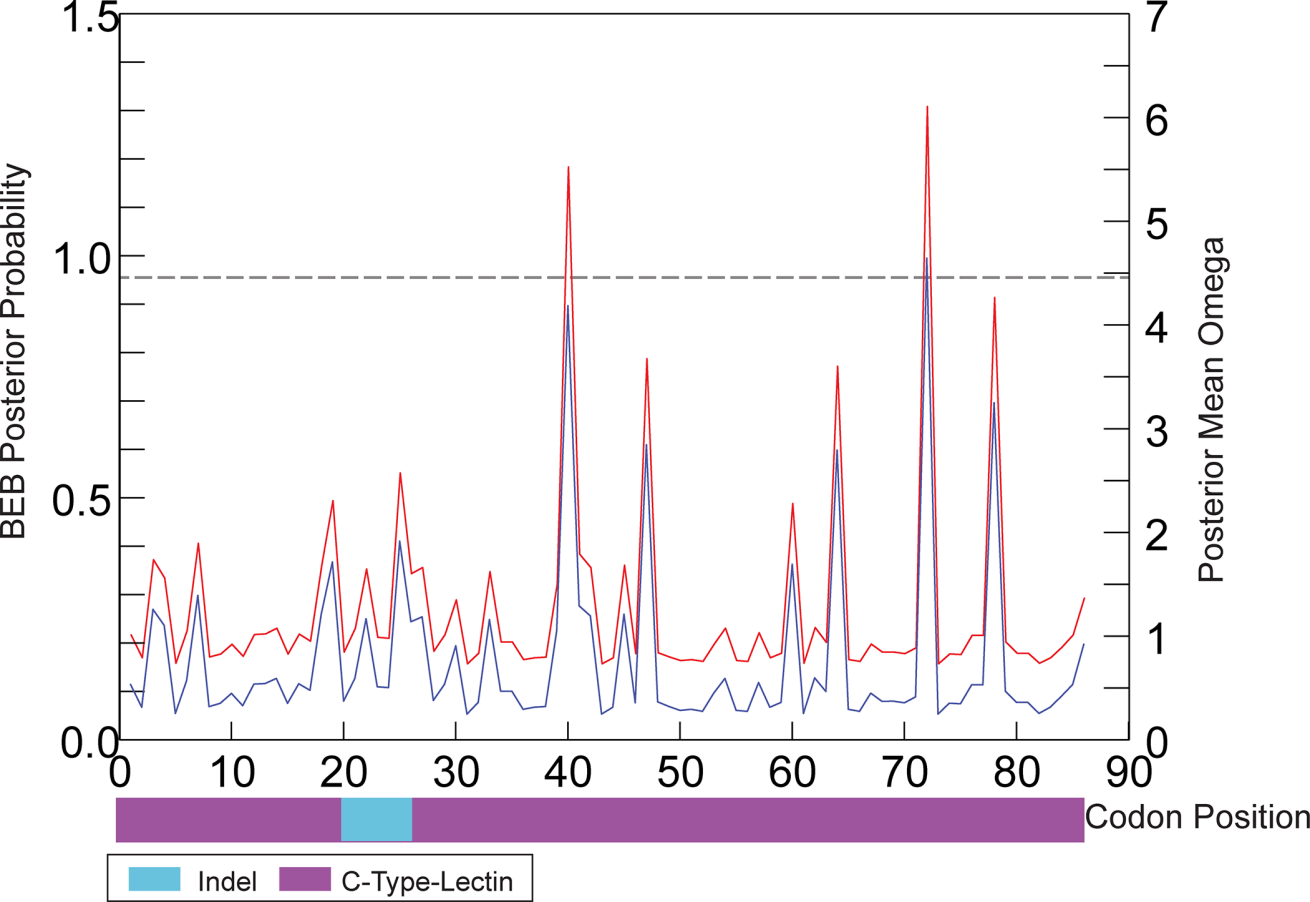
Positive selection within *Acp3*. Red line represents the mean posterior omega, and the blue line represents the probability of each codon being under positive selection. The values were calculated using a Bayes Empirical Bayes analysis under the M8 site-specific model in Paml. Codon position based on full-length alignment. The annotations identified using InterProScan and the position of the 24 bp indel are shown in relation to codon position.

A Bayes Empirical Bayes computation [101] implemented in PAML was used to assess the significance of the ω ratio at each codon position. Under the M8 model five sites were assigned to the positively selected class (ω >1), only one of which had a probability > 95% (ω = 6.366, Table 5, Fig 5). Observed changes at three of the five sites were non-conservative (S7 Table). It is acknowledged that likelihood-based methods can produce high levels of false positives [102, 103], however the alternative parsimony-based models tend to be very conservative and have low power detecting true positives, particularly in small datasets such as this one [104, 105]. The five sites presented here represent testable hypotheses for functionally important regions under selection within *Acp3* that could be examined with a larger dataset and other analysis methods. However, it should be noted that the function of *Acp3* is unknown, as is the exact position of these sites within the overall protein structure.

## Conclusions

Here we present the first male reproductive transcriptomes for *H*. *crassidens* and *H*. *thoracica*, resulting in putative gene sets of 1,754 and 2,691 non-redundant gene sets, respectively. We identified 865 putative reproductive associated proteins, and 113 orthologs, from which nine candidates were used for downstream evolutionary analyses. Our results suggest that positive selection may be acting on some *Hemideina* SFPs; in contrast, we were unable to detect positive selection on the general metabolic control genes. The lectin-related *Acp3* gene shows evidence for selection acting along the gene as a whole and on particular amino acids. This presents a testable hypothesis into what selection may be occurring on weta reproductive proteins. A better understanding can be achieved by incorporating functional and population genomics with candidate gene approaches to reveal the relationship between the evolution of these genes and mate recognition and speciation. In addition, the transcriptome data generated represents a first step in the identification of reproductive associated proteins in weta. These transcriptomic sequences will provide a valuable resource for further research into the evolution of reproductive proteins and speciation of New Zealand weta.

## Supporting information

S1 Table Sample collection details. **Samples used for 454 sequencing.
(XLSX)

S2 Table Primer sequences for candidate genes. Annotations from tblastx against non-redundant database.
(XLSX)

S3 Table Orthologous contigs pairs identified in the bidirectional tblastx search. (XLSX)

S4 Table Contigs identified in the reproductive protein search. (XLSX)

S5 Table McDonald Kreitman test results for each gene. ^a^ Yates correction is applied to G tests.
(XLSX)

S6 Table Likelihood values and parameter estimates for site-specific models for each gene. Parameters in brackets are not free, and therefore are not counted when considering difference in the number of parameters for nested model comparisons.
(XLSX)

S7 Table Sites identified as putatively under selection and their posterior probabilities under the M3 and M8 models. ^a^ Amino acid site in the original alignment, including gaps; BEB, Bayes Empirical Bayes posterior probability; NEB, Naïve Empirical Bayes; **Probability > 99%.
(XLSX)

S1 Fig Top-Hit species distribution for tblastx results for A) *Hemideina thoracica* and B) *Hemideina crassidens*.
(PDF)

S2 Fig Gene ontology categories identified in the reproductive gene screen for A) *Hemideina thoracica* and B) *Hemideina crassidens*.
(PDF)

S1 File *Deinacrida fallai* and *D*. *mahoenui* orthologues identified from an unpublished *de novo* assembled illumina transcriptome data. (FASTA)
