# Supplementary material for: Positive selection and comparative molecular evolution of reproductive proteins from New Zealand tree weta (Orthoptera, Hemideina)
Source: PLoS One. 2017 Nov 13;12(11):e0188147. doi: 10.1371/journal.pone.0188147 (PMC5683631; doi:10.1371/journal.pone.0188147)
Supplement: S1 Fig — Top-Hit species distribution for tblastx results for A) Hemideina thoracica and B) Hemideina crassidens. (PDF) [file pone.0188147.s008.pdf]

A)

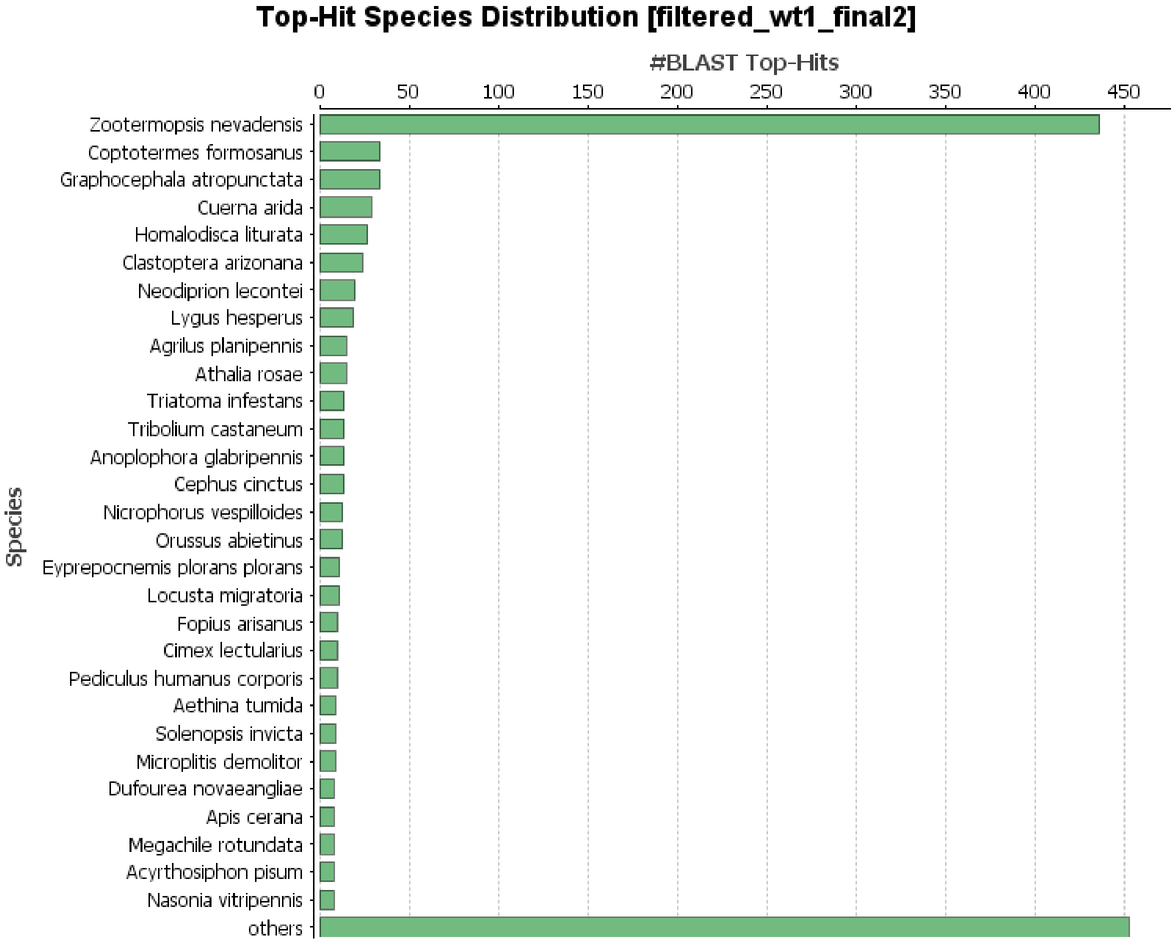
Top-Hit Species Distribution [filtered_wt1_final2]
#BLAST Top-Hits
0
50
100
150
200
250
300
350
400
450
Species
Zootermopsis nevadensis
Coptotermes formosanus
Graphocephala atropunctata
Cuerna arida
Homalodisca liturata
Clastoptera arizonana
Neodiprion lecontei
Lygus hesperus
Agrilus planipennis
Athalia rosae
Triatoma infestans
Tribolium castaneum
Anoplophora glabripennis
Cephus cinctus
Nicrophorus vespilloides
Orussus abietinus
Eyprepocnemis plorans plorans
Locusta migratoria
Fopius arisanus
Cimex lectularius
Pediculus humanus corporis
Aethina tumida
Solenopsis invicta
Microplitis demolitor
Dufourea novaeangliae
Apis cerana
Megachile rotundata
Acyrthosiphon pisum
Nasonia vitripennis
others

B)

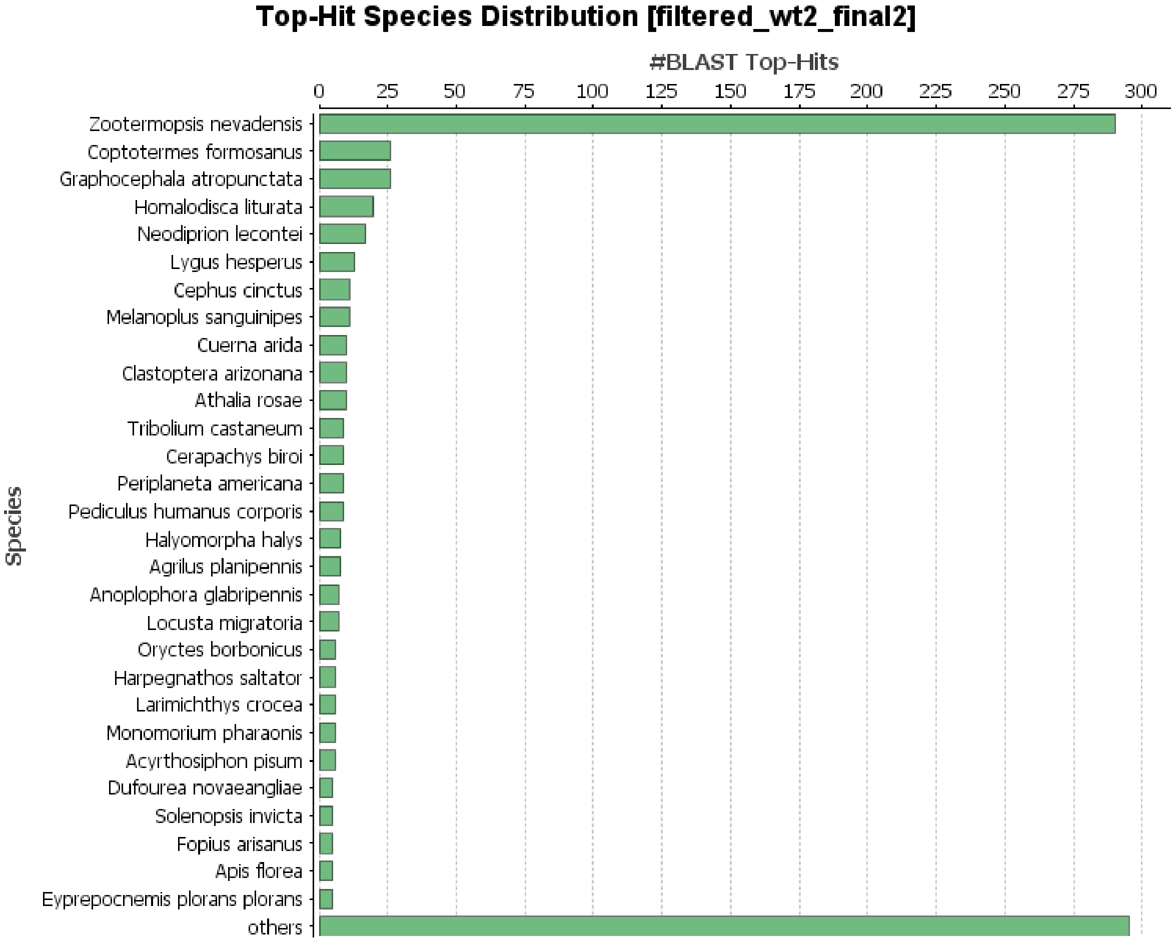
Top-Hit Species Distribution [filtered_wt2_final2]
#BLAST Top-Hits
0
25
50
75
100
125
150
175
200
225
250
275
300
Species
Zootermopsis nevadensis
Coptotermes formosanus
Graphocephala atropunctata
Homalodisca liturata
Neodiprion lecontei
Lygus hesperus
Cephus cinctus
Melanoplus sanguinipes
Cuerna arida
Clastoptera arizonana
Athalia rosae
Tribolium castaneum
Cerapachys biroi
Periplaneta americana
Pediculus humanus corporis
Halyomorpha halys
Agrilus planipennis
Anoplophora glabripennis
Locusta migratoria
Oryctes borbonicus
Harpegnathos saltator
Larimichthys crocea
Monomorium pharaonis
Acyrthosiphon pisum
Dufourea novaeangliae
Solenopsis invicta
Fopius arisanus
Apis florea
Eyprepocnemis plorans plorans
others
