# Supplementary figures and images for: Positive selection and comparative molecular evolution of reproductive proteins from New Zealand tree weta (Orthoptera, Hemideina)
Source: PLoS One. 2017 Nov 13;12(11):e0188147. doi: 10.1371/journal.pone.0188147 (PMC5683631; doi:10.1371/journal.pone.0188147)

A)

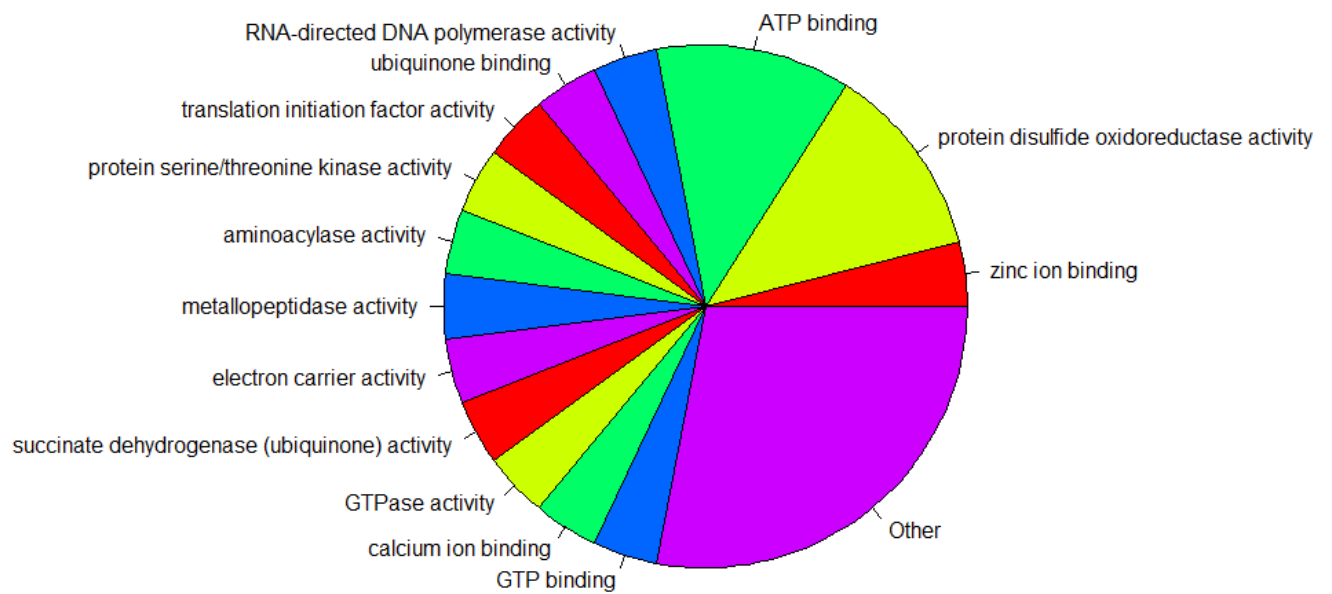

B)

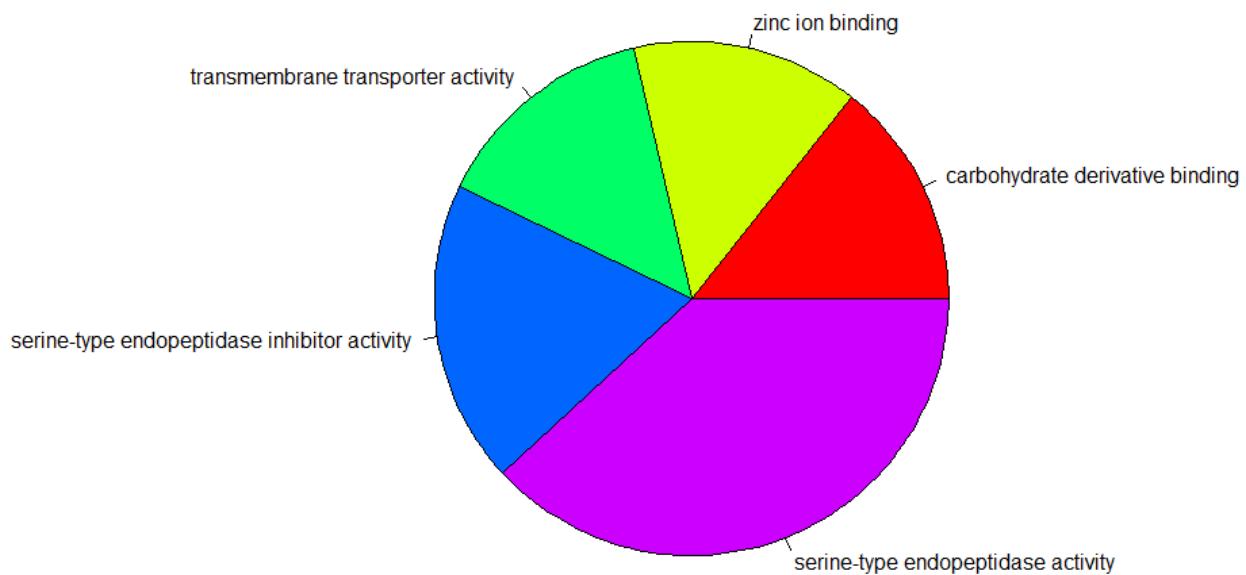

Supplement: S2 Fig — Gene ontology categories identified in the reproductive gene screen for A) Hemideina thoracica and B) Hemideina crassidens. (PDF) [file pone.0188147.s009.pdf]
